# Long-lasting reduction in clonogenic potential of colorectal cancer cells by sequential treatments with 5-azanucleosides and topoisomerase inhibitors

**DOI:** 10.1186/s12885-016-2925-6

**Published:** 2016-11-16

**Authors:** Alicja Pawlak, Ewa Ziolo, Anna Fiedorowicz, Klaudyna Fidyt, Leon Strzadala, Wojciech Kalas

**Affiliations:** 1 Hirszfeld Institute of Immunology and Experimental Therapy, Polish Academy of Sciences, Rudolfa Weigla 12, 53-114 Wroclaw, Poland; 2 Jan Dlugosz University in Czestochowa, Waszyngtona 4/8, 42-200 Czestochowa, Poland

**Keywords:** 5-azacytidine, Akt, combinatorial therapy, Decitabine, DNA methylation, Doxorubicin, epigenetics, Etoposide, Irinotecan, Mitoxantrone

## Abstract

**Background:**

The currently approved therapies fail in a substantial number of colorectal cancer (CRC) patients due to the molecular heterogeneity of CRC, hence new efficient drug combinations are urgently needed. Emerging data indicate that 5-azanucleosides are able to sensitize cancer cells to the standard chemotherapeutic agents and contribute to overcoming intrinsic or acquired chemoresistance.

**Methods:**

CRC cells with different genetic backgrounds (HCT116, DLD-1, HT-29) were sequentially treated with 5-azanucleosides and topoisomerase inhibitors. The combined effects of these two drug classes on cell viability, apoptosis, signaling pathways, and colony formation were investigated.

**Results:**

Here, we demonstrate that pretreatment with DNA demethylating agents, 5-aza-2′-deoxycytidine and 5-azacytidine, sensitizes CRC cells to topoisomerase inhibitors (irinotecan, etoposide, doxorubicin, mitoxantrone), reducing cell viability and clonogenicity and increasing programmed cell death more effectively than individual compounds at the same or even higher concentrations. 5-Azanucleosides did not cause considerable immediate toxic effects as evaluated by analysis of cell viability, apoptosis, DNA damage (γH2A.X), and endoplasmic reticulum (ER) stress (CHOP). However, 5-azanucleosides exerted long-lasting effects, reducing cell viability, changing cell morphology, and affecting phosphoinositide 3-kinase (PI3-kinase)/Akt signaling pathway. We found that a single exposure to 5-azanucleosides is sufficient to induce long-lasting sensitization to topoisomerase inhibitors. The combinatorial, but not separate, treatment with low doses of 5-aza-2′-deoxycytidine (0.1 μM) and etoposide (0.5 μM) caused a long-lasting (almost 70 days) reduction in clonogenic/replating ability of DLD-1 cells.

**Conclusions:**

These results suggest that sequential treatments with DNA demethylating agents and topoisomerase inhibitors may exert clinically relevant anticancer effects.

**Electronic supplementary material:**

The online version of this article (doi:10.1186/s12885-016-2925-6) contains supplementary material, which is available to authorized users.

## Background

Colorectal cancer (CRC) was the third most common cancer worldwide with 1.36 million new cases and nearly 0.7 million deaths in 2012 [1]. Currently, 5-fluorouracil, irinotecan (also known as CPT-11), and oxaliplatin constitute the backbone of chemotherapy for CRC. Unfortunately, the molecular heterogeneity of CRC creates the considerable variability in response to treatment among patients with the same disease stage. Because the currently approved therapies fail in a substantial number of CRC patients, new efficient drug combinations are constantly being sought.

It is now well established that genetic mutations cooperate with epigenetic changes to drive the formation and progression of normal colorectal epithelium into adenocarcinomas. Abnormalities in DNA methylation occur early in cancer progression, even before the appearance of the aberrant crypt foci (ACF), the first neoplastic lesions identified in CRC formation [2, 3]. Cancer cells are characterized by genome-wide hypomethylation compared to normal cells leading to chromatin architecture reorganization, genomic instability, loss of imprinting, and activation of oncogenes. On the other hand, local hypermethylation of CpG islands in the promoter regions of certain genes contributes to their transcriptional inactivation [4]. It was observed that among these silenced genes are many well-characterized tumor suppressor genes [3, 5]. Hypermethylation of tumor suppressor genes is assumed to be functionally equivalent to genetic loss-of-function mutations.

The reversibility of epigenetic modifications makes them attractive targets for cancer treatment. The two most extensively studied inhibitors of DNA methylation are 5-azacytidine (5-aza-C) and 5-aza-2′-deoxycytidine (5-aza-dC, decitabine), analogues of cytidine and 2′-deoxycytidine, respectively, with a nitrogen atom replacing the carbon at the five position of the cytosine base [6]. These compounds were first synthesized in the 1960s and were initially tested against acute myeloid leukemia (AML). In 2004 (5-aza-C) and 2006 (5-aza-dC) they were approved by the U.S. Food and Drug Administration (FDA) for the treatment of patients with myelodysplastic syndromes (MDS) [7, 8]. Once incorporated into the genome during replication as regular nucleotides, 5-aza-dC and 5-aza-C (from now referred to as 5-azanucleosides) that replace cytosine targets for methylation form irreversible complexes with DNA (cytosine-5) methyltransferases (DNMTs) leading to the depletion of enzymes and passive loss of cytosine methylation with each round of cell division. This results in DNA hypomethylation and reexpression of silenced tumor suppressor genes, which were inactivated during carcinogenesis, thereby restoring proliferation control and apoptosis sensitivity [9]. It was also reported that 5-azanucleosides induce selective and replication-independent degradation of DNMT1 by a proteasomal pathway [10].

Emerging data indicate that 5-azanucleosides are able to sensitize cancer cells to the standard chemotherapeutic agents and contribute to overcoming intrinsic or acquired chemoresistance [11]. For instance, 5-azanucleosides have been shown to increase the sensitivity of cancer cells to cisplatin (prostate cancer, neuroblastoma), erlotinib and/or gefitinib (AML, CRC), docetaxel (prostate cancer), doxorubicin (breast cancer, neuroblastoma), etoposide (neuroblastoma), and carboplatin (ovary cancer) [12–17].

In the present study, we demonstrated that DNA demethylating agents (5-aza-dC, 5-aza-C) induce long-lasting sensitization of CRC cells to inhibitors of topoisomerase I (irinotecan) and topoisomerase II (etoposide, doxorubicin, mitoxantrone). The results of our study may contribute to improving the effectiveness of current treatments for CRC and possibly other cancers.

## Methods

### Cell culture and treatment

The human colorectal carcinoma cell lines HCT116, DLD-1, DKs-8, and HT-29 were maintained in high-glucose (4.5 g/l; POCH) DMEM (Hirszfeld Institute of Immunology and Experimental Therapy, Polish Academy of Sciences) supplemented with 10% FBS (Gibco, Thermo Fisher Scientific) and Antibiotic Antimycotic Solution (Sigma-Aldrich). All cell lines were cultured at 37 °C in a humidified atmosphere of 5% CO_2_. The cells were seeded at densities of 4 × 10^3^ cells/0.1 ml (0.32 cm^2^) (cell viability assay), 6 × 10^3^–1 × 10^4^ cells/0.3 ml (0.7 cm^2^) (microscopic images), 2 × 10^4^ cells/0.5 ml (1.9 cm^2^) (flow cytometry), 1 × 10^5^ cells/3 ml (9.5 cm^2^) (long-term colony formation assay, serial replating assay), 3.5 × 10^5^ cells/3–4 ml (21 cm^2^) (Western blotting). In short-term experiments, the cells were treated either with 5-aza-dC (1 μM; Cayman Chemical) or 5-aza-C (4 μM; Cayman Chemical) at the day of seeding. After 2 days, the culture medium was changed and the cells were treated either with 5-aza-dC or 5-aza-C along either with irinotecan (5–75 μM; Cayman Chemical), etoposide (5–50 μM; Sigma-Aldrich), doxorubicin (0.05–0.9 μM; Cayman Chemical), or mitoxantrone (0.05–1 μM; Cayman Chemical). Two or 3 days later, the cells were collected for an appropriate assay. In long-term colony formation assay and serial replating assay, the cells were treated with 5-aza-dC (0.1 μM) 1 day post-seeding. The next day, the cells were treated with etoposide (0.1–1 μM) and collected after two more days. In Western blotting analyses of Akt, phospho-Akt, mTOR, phospho-mTOR, p70S6K, and phospho-p70S6K expression, the cells were treated with 5-aza-dC (0.1–1 μM) 1 day post-seeding. The cells were maintained in culture medium without 5-aza-dC (the first passage after 3 days) and collected after six and twenty days. All compounds were dissolved in DMSO (Sigma-Aldrich). Further dilutions were made in culture medium immediately prior to experiment. The final DMSO concentration never exceeded 0.75%.

### Cell viability assay

Cell viability was assessed by CellTiter 96 AQ_ueous_ One Solution Cell Proliferation Assay (Promega) according to the manufacturer’s protocol. Each treatment within a single experiment was performed in triplicate. Absorbance at 490 nm was recorded by a Wallac 1420 VICTOR^2^ plate reader (PerkinElmer, Waltham, MA, USA). Data were normalized to untreated control. The nature of the interactions between 5-azanucleosides and topoisomerase inhibitors was analyzed by the Chou-Talalay method [18, 19] using CompuSyn software (ComboSyn, Paramus, NJ, USA). Combination Index (CI) values for each drug combination were determined as a quantitative measure of drug-drug interaction. A CI value of < 0.9 indicates synergy, a CI value of 0.9–1.1 indicates additive effect, and a CI value of > 1.1 indicates antagonism.

### Microscopic images

The cells were fixed with 4% paraformaldehyde/PBS (Sigma-Aldrich) for 30 min at 37 °C and washed three times with PBS (with Ca^2+^ and Mg^2+^). The microscopic images were taken using an Olympus IX-81 inverted fluorescence microscope (Olympus, Tokyo, Japan) with a 10x objective and analyzed using cellSens software (Olympus).

### Flow cytometry

Both attached and detached cells were collected for DNA fragmentation analyses. The cells were fixed with ice-cold 70% ethanol (POCH) for 30 min at 4 °C and stained with PI/PBS (50 μg/ml; Sigma-Aldrich) in the presence of RNase A (0.02 mg/ml; Sigma-Aldrich) overnight at 4 °C in the dark. In between steps, the cells were washed with cold PBS (with Ca^2+^ and Mg^2+^) containing 2.5% FBS. Apoptosis was also assessed by Annexin V Apoptosis Detection Kit (Santa Cruz Biotechnology) according to the manufacturer’s protocol. Briefly, the cells were stained with Annexin V-FITC (8 μg/ml) and PI (5 μg/ml) for 15 min at RT in the dark. Data was acquired on a FACSCalibur flow cytometer (Becton Dickinson, Franklin Lakes, NJ, USA) and analyzed using Flowing Software 2.5.1 software (Perttu Terho, Turku, Finland). Apoptosis was quantified as percentage of cells with a hypodiploid DNA content (sub-G_1_ cell population) and percentage of both Annexin V-positive and Annexin V/PI-double-positive cells.

### Long-term colony formation assay and serial replating assay

The viable cells were counted using a hemacytometer (trypan blue exclusion method) and seeded in duplicates at a density of 5 × 10^2^ cells/6 ml (21 cm^2^). The dishes had been pre-coated with poly-L-lysine/PBS (0.001%; Sigma-Aldrich) and washed twice with PBS (with Ca^2+^ and Mg^2+^). After 2 weeks, the colonies were fixed and stained with 1% crystal violet/ethanol (Sigma-Aldrich), documented with an Olympus Stylus SH-50 camera (Olympus), and counted manually using ImageJ 1.47 software (National Institutes of Health, Bethesda, MD, USA). In serial replating assay, several colonies from the first plating were collected, cultured, and seeded in the second plating. After 2 weeks, several colonies from the second plating were collected, cultured, and seeded in the third plating. The term plating efficiency (PE) indicates the percentage of seeded cells that grow to form colonies. The surviving fraction (SF) is calculated as a ratio between PEs of treated and control cells multiplied by 100.

### Western blotting

Both attached and detached cells were collected for H2A.X, γH2A.X, and CHOP expression analyses, whereas only attached cells were taken for Akt, phospho-Akt, mTOR, phospho-mTOR, p70S6K, and phospho-p70S6K expression analyses. Whole cell lysates were prepared using cold RIPA buffer [150 mM NaCl (POCH), 50 mM Tris-HCl pH 8.0 (BioShop Canada), 1% NP-40 (Calbiochem), 0.5% sodium deoxycholate (Sigma-Aldrich), 1% SDS (BioShop Canada)] supplemented with Sigma*FAST* Protease Inhibitor Cocktail (Sigma-Aldrich) and Halt Phosphatase Inhibitor Cocktail (Thermo Fisher Scientific). The cell lysates were then sonicated for 10 s at 100% power using a Sonopuls HD 2070 ultrasonic homogenizer (Bandelin, Berlin, Germany) and centrifuged at 10,000 x g for 10 min at 4 °C to pellet cellular debris. Protein concentration was determined by Pierce BCA Protein Assay Kit (Thermo Fisher Scientific) according to the manufacturer’s protocol. Absorbance at 570 nm was recorded by a Wallac 1420 VICTOR^2^ plate reader. Cell lysates with Laemmli sample buffer [50 mM Tris-HCl pH 6.8, 10% glycerol (BioShop Canada), 5% 2-mercaptoethanol (Sigma-Aldrich), 2% SDS, 0.05% bromophenol blue (BioShop Canada)] were heated for 5 min at 95 °C, the proteins were separated by SDS-PAGE as described by Laemmli [20] using 8–15% resolving gels [SDS-PAGE running buffer: 25 mM Tris, 192 mM glycine (BioShop Canada), 0.1% SDS] and transferred (semi-dry transfer) to PVDF membrane (0.45 μm pore size; Merck Millipore) [transfer buffer: 25 mM Tris, 192 mM glycine, either 10% or 20% methanol (POCH)]. In between steps, membranes were washed with TBST [20 mM Tris, 150 mM NaCl, 0.1% Tween 20 (BioShop Canada)]. Membranes were blocked either with 1% casein [0.1 M Tris-HCl pH 8.0, 214 mM NaCl, 1% casein from bovine milk (Sigma-Aldrich)] or 5% BSA/TBST (Sigma-Aldrich) for an hour at RT or overnight at 4 °C and then incubated with primary antibody overnight at 4 °C. After probing with HRP-conjugated secondary antibody for 1 h at RT, proteins of interest were detected using SuperSignal West Dura Extended Duration Substrate (Thermo Fisher Scientific). The following antibodies were used in this study: anti-Akt (1:1000, #4691; Cell Signaling Technology), anti-phospho-Akt (1:1000, #4060; Cell Signaling Technology), anti-CHOP (1:2000, #2895; Cell Signaling Technology), anti-H2A.X (1:2000, #2595; Cell Signaling Technology), anti-γH2A.X (1:2000, #9718; Cell Signaling Technology), anti-mTOR (1:1000, #2983; Cell Signaling Technology), anti-phospho-mTOR (1:1000, #2974; Cell Signaling Technology), anti-p70S6K (1:1000, #2708; Cell Signaling Technology), anti-phospho-p70S6K (1:1000, #9234; Cell Signaling Technology), anti-actin/HRP (1:2000, #sc-1615; Santa Cruz Biotechnology), anti-mouse/HRP (1:2500, #P0447; Dako, Agilent Technologies), anti-rabbit/HRP (1:2000–3000, #P0048; Dako, Agilent Technologies).

### Statistical analysis

Data are presented as means ± SD of results from at least three independent experiments. Comparisons between two groups (DNA demethylating agent treatment group vs. combinatorial treatment group; topoisomerase inhibitor treatment group vs. combinatorial treatment group) were analyzed by two-tailed Student’s t-test. Significance was assumed at *p* < 0.05. The Median Absolute Deviation (MAD) test was employed to detect outliers. Asterisks in the figures indicate that a combinatorial treatment group was, at the same time, significantly different from a) DNA demethylating agent treatment group alone; AND b) topoisomerase inhibitor treatment group alone.

## Results

### 5-azanucleosides sensitize CRC cells to topoisomerase inhibitors

**Fig. 1:**
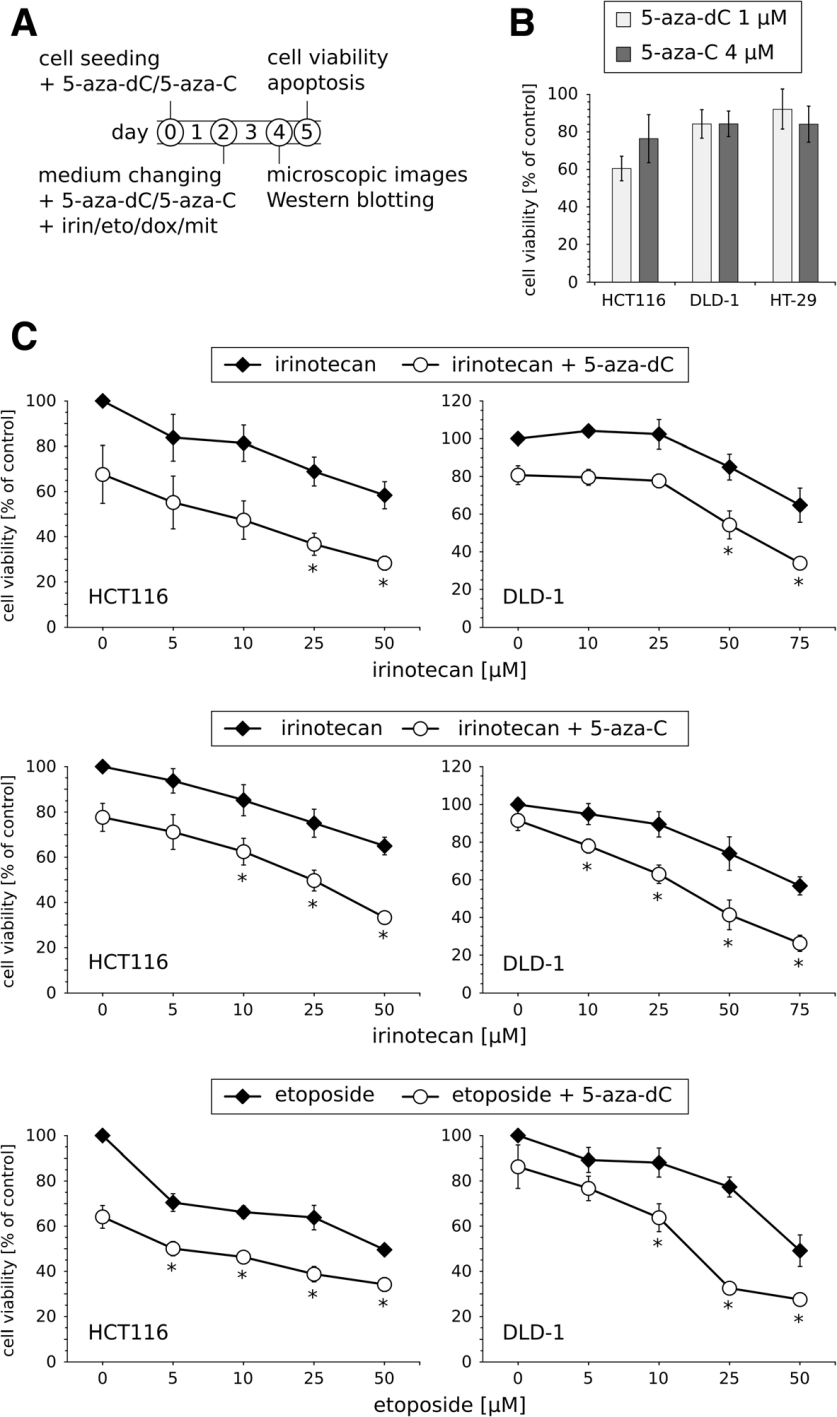
Pretreatment with 5-azanucleosides enhances the cytotoxicity of topoisomerase inhibitors in CRC cells. **a** Treatment scheme for short-term experiments. **b** Cell viability of HCT116, DLD-1, and HT-29 cells after exposure to 1 μM 5-aza-dC (*n* ≥ 14) or 4 μM 5-aza-C (*n* ≥ 8) alone. **c** Cell viability of HCT116 and DLD-1 cells after sequential treatments with 5-azanucleosides (1 μM 5-aza-dC or 4 μM 5-aza-C) and topoisomerase inhibitors (5–75 μM irinotecan or 5–50 μM etoposide). Data are presented as means ± SD normalized to untreated control. *P < 0.05 compared with DNA demethylating agent treatment group and topoisomerase inhibitor treatment group. Irin - irinotecan, eto - etoposide, dox - doxorubicin, mit - mitoxantrone

**Fig. 2:**
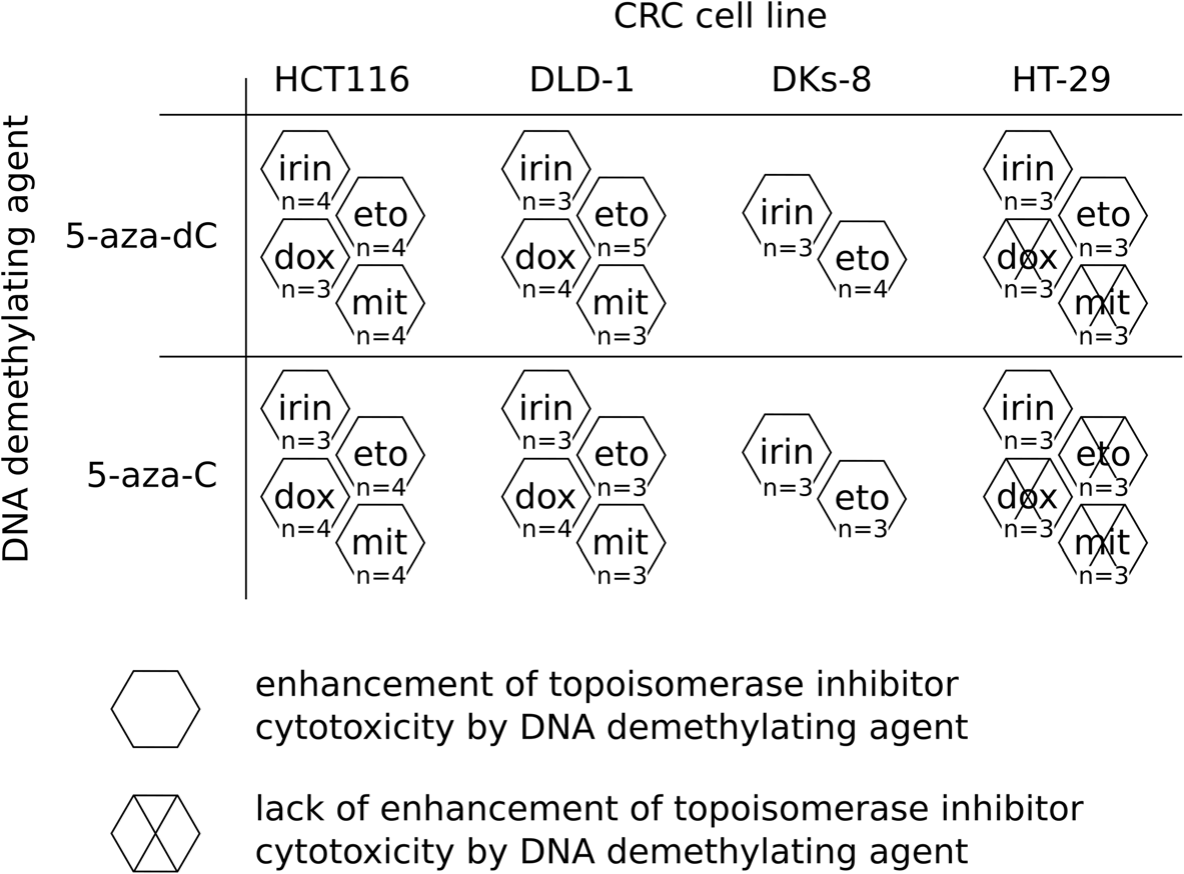
Schematic summary of cell viability results. They demonstrate that pretreatment with 5-azanucleosides can reinforce the effectiveness of most tested topoisomerase inhibitors in all tested CRC cell lines. Crossed hexagons indicate lack of synergy between a DNA demethylating agent and a topoisomerase inhibitor (based on CI values calculated by the Chou-Talalay method). The number of independent experiments (n value) for each combination in each cell line was given. All the data are presented in details in Additional file 1: Table S1. Irin - irinotecan, eto - etoposide, dox - doxorubicin, mit - mitoxantrone

In order to examine the mutual effects of DNA demethylating agents and topoisomerase inhibitors, we analyzed the viability of CRC cells after sequential treatments with 5-azanucleosides (5-aza-dC, 5-aza-C) and inhibitors of topoisomerase I (irinotecan) or topoisomerase II (etoposide, doxorubicin, mitoxantrone). In our study, we used the well-established CRC cell lines with different genetic backgrounds: HCT116, DLD-1, HT-29 (Table 1). The cells were treated with 5-aza-dC (1 μM) or 5-aza-C (4 μM) for 48 h followed by culture medium change and a second treatment with 5-azanucleosides in combination with irinotecan (5–75 μM), etoposide (5–50 μM), doxorubicin (0.05–0.9 μM), or mitoxantrone (0.05–1 μM) (Fig. 1a). 5-Aza-dC and 5-aza-C alone decreased cell viability by an average of 39.6 and 23.8% in HCT116 cells, 15.9 and 15.9% in DLD-1 cells, 8.1 and 16.0% in HT-29 cells, respectively (Fig. 1b). In all tested cell lines, the combinatorial treatments reduced cell viability more effectively than those of individual compounds at the same or even higher concentrations (Fig. 1c, Fig. 2, Additional file 1: Table S1). For instance, in HCT116 cells, the combinatorial treatment with 5-aza-dC and 25 μM irinotecan was more effective than 50 μM irinotecan used alone. All the data, along with Combinatorial Index (CI) values calculated by the Chou-Talalay method, are summarized in Fig. 2 and presented in details in Additional file 1: Table S1. They indicate that pretreatment with 5-azanucleosides was able to reinforce (in a synergistic manner) the effectiveness of most tested topoisomerase inhibitors in all tested cell lines. More specifically, 5-aza-dC and 5-aza-C pretreatment made HCT116 and DLD-1 cells sensitive to all topoisomerase inhibitors used, whereas 5-azanucleosides-treated HT-29 cells had an increased sensitivity to irinotecan, and in the case of 5-aza-dC, also to etoposide.

**Table 1:** Mutational status of colorectal cancer critical genes [44, 51–55]

CRC cell line	Oncogenes		Tumor suppressor genes	
	KRAS	BRAF	TP53	MLH1
HCT116	G13D / wt	wt / wt	wt / wt	S252* / S252* ^a^
DLD-1	G13D / wt	wt / wt	S241F / wt (sil)	wt / wt ^b^
DKs-8	- / wt	wt / wt	S241F / wt (sil)	wt / wt ^b^
HT-29	wt / wt	V600E / wt	R273H / R273H	wt (m) / wt ^c^

*Wt* wild-type; *sil* silenced; *m* methylated; *nonsense mutation

^a^HCT116 cells are MMR-deficient due to mutations in *MLH1* and mutS homolog 3 (*MSH3*) genes

^b^DLD-1 and DKs-8 cells are MMR-deficient due to mutation in mutS homolog 3 (*MSH6*) gene

^c^HT-29 cells are MMR-proficient

CRC driving mutations may influence cancer susceptibility to chemotherapy [21–23]. We examined the relevance of a KRas mutation using the DLD-1/DKs-8 isogenic cell line pair, where DLD-1 cells have activating mutation in KRas (p.G13D) and the mutant allele was disrupted in DKs-8 cells (Table 1). The sequential treatment with 5-aza-dC and etoposide (Additional file 2: Figure S1), as well as other combinations with 5-azanucleosides and topoisomerase inhibitors (Fig. 2, Additional file 1: Table S1), decreased DKs-8 cell viability compared to each compound alone. Similar inhibitory effects were observed in DLD-1 cells, which indicates the negligible role of KRas mutational status in mutual actions of 5-azanucleosides and topoisomerase inhibitors in CRC cells.

### Sequential treatments with 5-azanucleosides and topoisomerase inhibitors increase apoptosis in CRC cells, but not through enhancement of DNA damage or ER stress

**Fig. 3:**
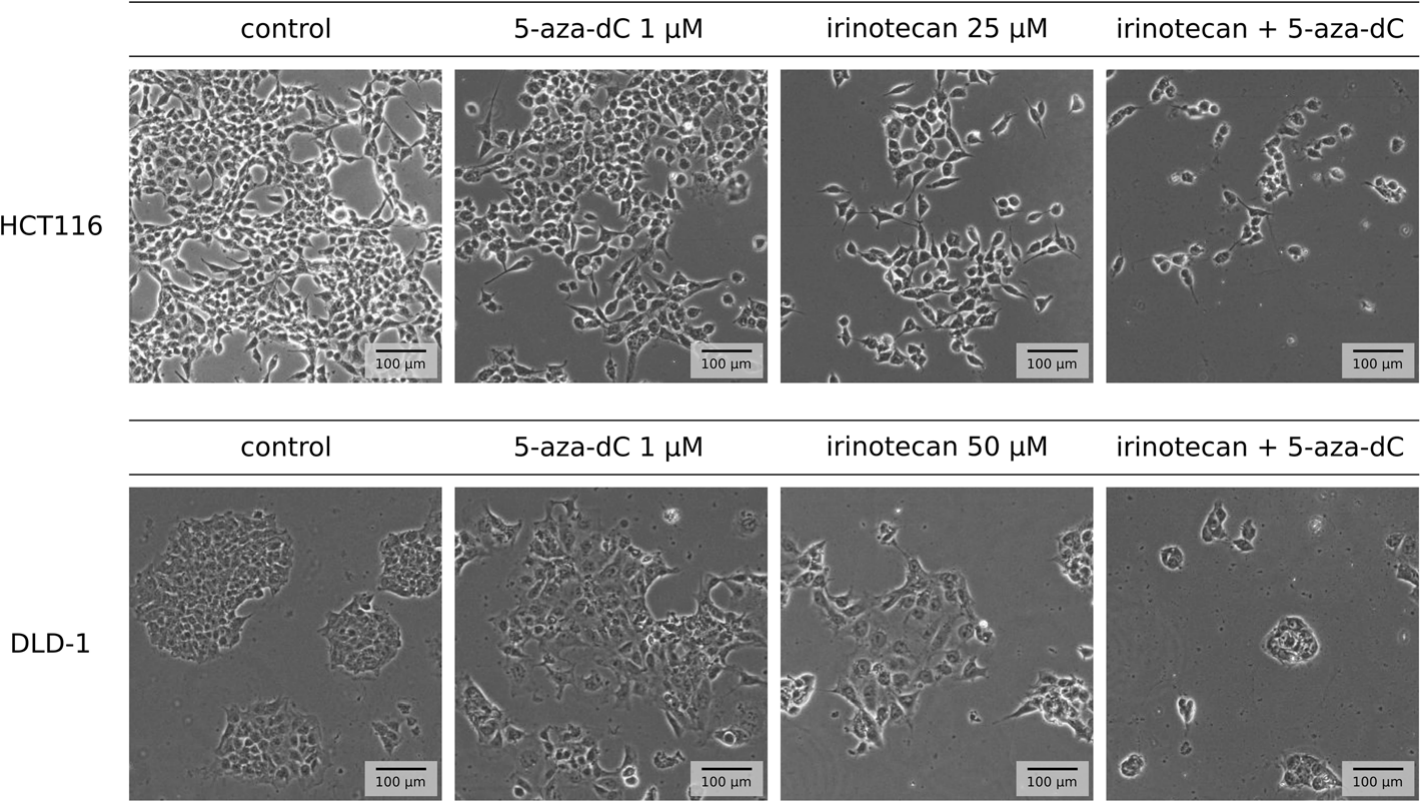
Combinatorial treatments cause changes in CRC cell morphology. Representative bright-field microscopic images (bar = 100 μm) of HCT116 and DLD-1 cells after sequential treatments with 1 μM 5-aza-dC and 25-50 μM irinotecan. Figure 1a shows the treatment scheme

**Fig. 4:**
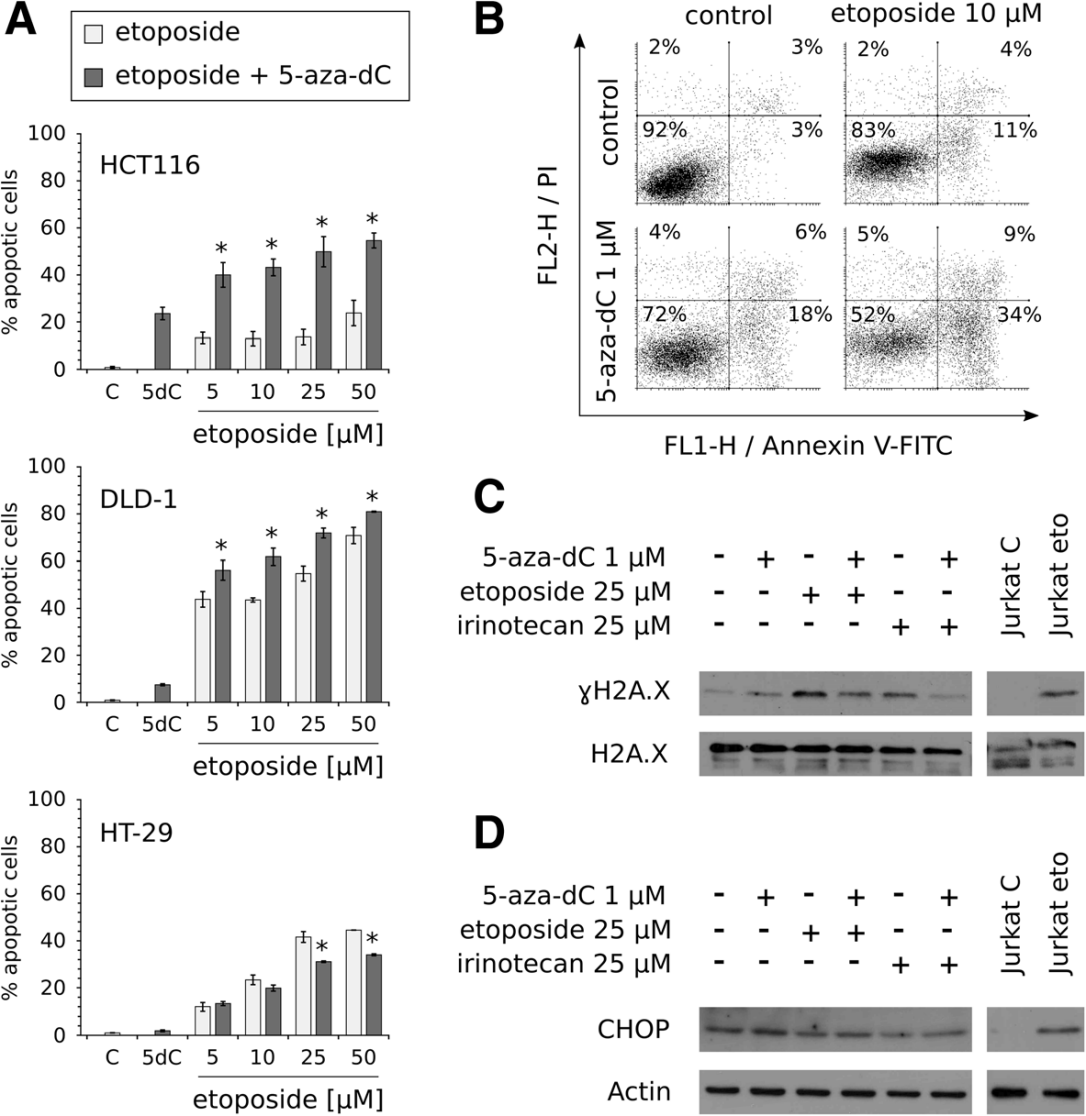
Combinatorial treatments increase CRC cell apoptosis, but not through DNA damage or ER stress enhancement. **a** Apoptosis-associated DNA fragmentation of HCT116, DLD-1, and HT-29 cells after sequential treatments with 1 μM 5-aza-dC and 5–50 μM etoposide (*n* = 3). Figure 1a shows the treatment scheme. Data are presented as means ± SD. *P < 0.05 compared with DNA demethylating agent treatment group and topoisomerase inhibitor treatment group. Representative histograms are presented in Additional file 3: Figure S2. **b** Representative Annexin V-FITC/PI-double staining histograms of HCT116 cells after sequential treatments with 1 μM 5-aza-dC and 10 μM etoposide. Figure 1a shows the treatment scheme. More results are presented in Additional file 4: Figure S3. **c** Representative immunoblot of γH2A.X (Ser139) expression from HCT116 cells sequentially treated with 1 μM 5-aza-dC and 25 μM etoposide/irinotecan (*n* = 3). H2A.X served as a loading control. **d** Representative immunoblot of CHOP expression from HCT116 cells sequentially treated with 1 μM 5-aza-dC and 25 μM etoposide/irinotecan (*n* = 4). Actin served as a loading control. Figure 1a shows the treatment scheme. Jurkat cells treated with 25 μM etoposide served as a positive control. C - control, eto - etoposide

The described decreases in CRC cell viability after sequential treatments with 5-azanucleosides and topoisomerase inhibitors resulted from a reduction in cell number (Fig. 3, data not shown). The combinatorial treatments caused changes in cell morphology, e.g. HCT116 cells were less elongated, whereas DLD-1 cells were less densely packed than control and irinotecan- or 5-aza-dC-treated cells (Fig. 3). Furthermore, there were more floating cells in culture medium. To examine whether cell number reduction was attributed to enhanced cell death, we measured apoptosis-associated DNA fragmentation by staining cells with propidium iodide (PI). 5-Aza-dC alone increased the number of cells with a hypodiploid DNA content to 23.6 ± 2.7% in HCT116 cells, 7.5 ± 0.4% in DLD-1 cells, and 2.0 ± 0.4% in HT-29 cells (Fig. 4a, Additional file 3: Figure S2). The percentage of apoptotic cells increased in a concentration-dependent manner after etoposide treatment. Regardless of whether the cells were resistant (HCT116) or sensitive (DLD-1) to etoposide-induced apoptosis, pretreatment with 5-aza-dC increased the number of apoptotic cells more effectively than those of individual compounds at the same or even higher concentrations. For instance, in HCT116 cells, the combinatorial treatment with 5-aza-dC and 5 μM etoposide was more effective than 50 μM etoposide used alone, showing the potential of 5-azanucleosides to overcome resistance to topoisomerase inhibitors in CRC cells. Similar results were obtained with Annexin V-FITC/PI-double staining (Fig. 4b, Additional file 4: Figure S3). On the other hand, pretreatment with 5-aza-dC did not enhance etoposide-induced DNA fragmentation in HT-29 cells (Fig. 4a, Additional file 3: Figure S2). Instead, a slight decrease in the number of cells with a hypodiploid DNA content was observed. These results were not consistent with Annexin V-FITC/PI-double staining (Additional file 4: Figure S3), suggesting a non-apoptotic form of programmed cell death in HT-29 cells.

5-Azanucleosides have previously been shown to be able to induce DNA damage [24, 25]. In this regard, we investigated whether sequential treatments with 5-azanucleosides and topoisomerase inhibitors could cause DNA damage accumulation in CRC cells. Histone H2A.X phosphorylation on serine 139 (γH2A.X) is a widely used marker of DNA damage induced by DNA double-strand breaks formation. As shown by Western blotting analysis, 5-aza-dC alone only weakly induced γH2A.X in HCT116 cells (Fig. 4c). Etoposide and irinotecan alone induced H2A.X phosphorylation, but in combination with 5-aza-dC, γH2A.X levels were decreased compared to etoposide or irinotecan alone. Pretreatment with 5-aza-dC did not alter the levels of etoposide- and irinotecan-induced γH2A.X in DLD-1 cells (data not shown).

Since it was reported that zebularine, another cytidine analogue and DNA methylation inhibitor, induces endoplasmic reticulum (ER) stress in HCT116 cells [26], we examined the possibility that 5-azanucleosides sensitize CRC cells to topoisomerase inhibitors by increasing ER stress, thereby leading to cell death. As a marker of ER stress-mediated apoptosis we used CCAAT/enhancer-binding protein (C/EBP) homologous protein (CHOP). As shown by Western blotting analysis, etoposide and irinotecan alone, as well as their combinations with 5-aza-dC, did not induce CHOP expression neither in HCT116 (Fig. 4d) nor DLD-1 cells (data not shown).

### Single treatments with 5-azanucleosides exert long-lasting effects on HCT116 and DLD-1 cells

**Fig. 5:**
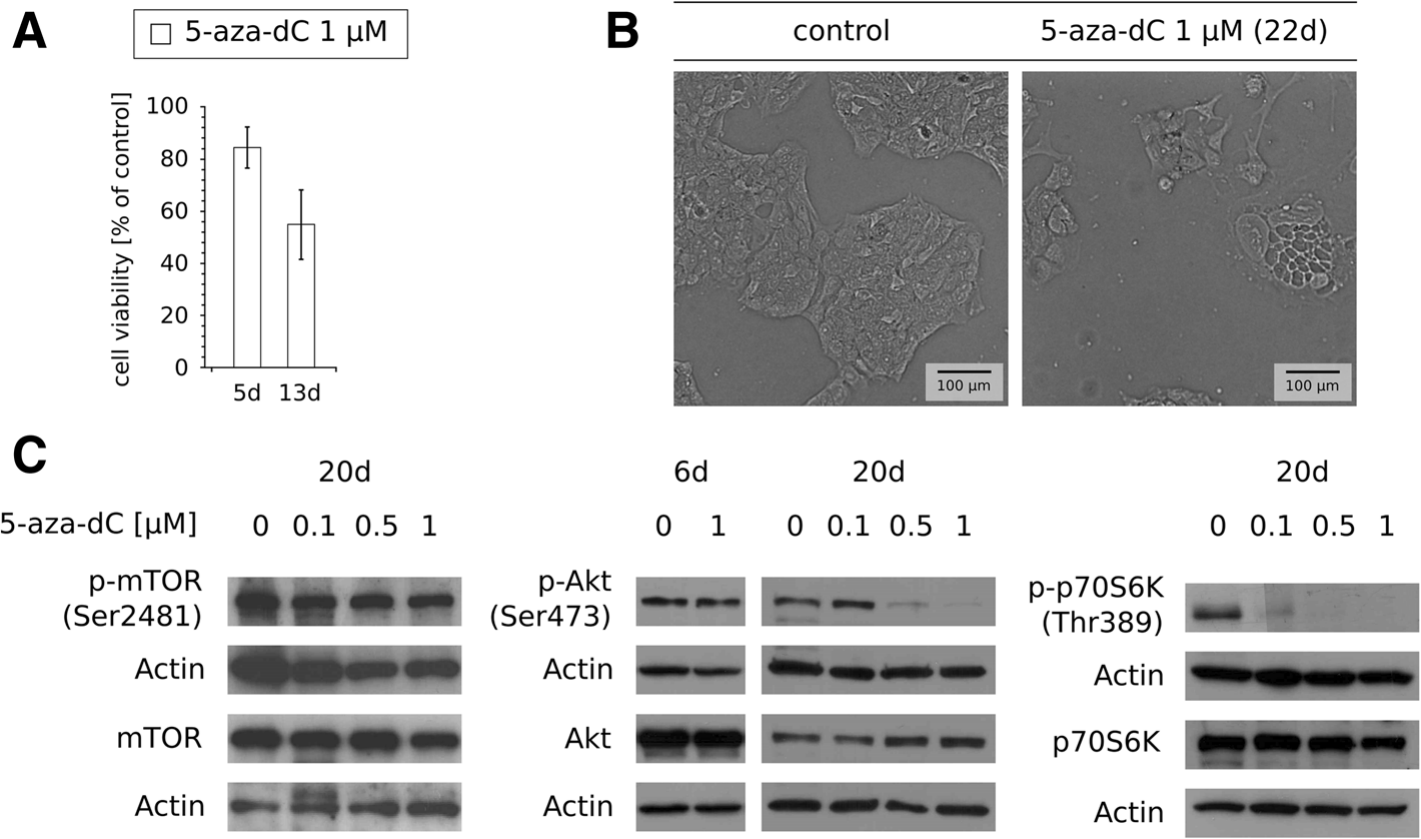
5-Aza-dC exerts long-lasting effects on CRC cells. **a** Cell viability of DLD-1 cells 5 (*n* = 15) and 13 (*n* = 4) days after exposure to 1 μM 5-aza-dC alone. Data are presented as means ± SD normalized to untreated control. **b** Representative bright-field microscopic images (bar = 100 μm) of DLD-1 cells 22 days after exposure to 1 μM 5-aza-dC alone. **c** Representative immunoblots of phospho-mTOR (Ser2481), mTOR, phospho-Akt (Ser473), Akt, phospho-p70S6K (Thr389), and p70S6K expression from DLD-1 cells 6 and 20 days after exposure to 0.1–1 μM 5-aza-dC alone (*n* ≥ 3). Actin served as a loading control. D - days

Having determined that 5-azanucleosides did not cause considerable immediate toxic effects on CRC cells as evaluated by analysis of cell viability (Fig. 1b, Additional file 1: Table S1), apoptosis (Fig. 4a, b, Additional file 3: Figure S2, Additional file 4: Figure S3), DNA damage (Fig. 4c), and ER stress (Fig. 4d), we presumed that 5-azanucleosides-induced sensitization to topoisomerase inhibitors could be attributed to epigenetic alterations, which can cause profound changes in cell homeostasis for a longer period of time. In this regard, we analyzed the viability of CRC cells at a later time point following a single treatment with 5-azanucleosides. Indeed, in DLD-1 cells, 5 days after 5-aza-dC treatment, cell viability decreased only by 15.9%, whereas 13 days after 5-aza-dC treatment, there was a 44.6% reduction in cell viability (Fig. 5a). According to the literature reports [27], we found that 5-azanucleosides reduce CRC proliferation (Additional file 5: Figure S4). The morphology of 5-aza-dC-treated cells was changing over time, reflecting the deterioration of their condition, despite the fact that the cells were regularly passaged (Fig. 5b). Similar effects were observed in HCT116 cells (data not shown), showing that 5-azanucleosides exert long-lasting effects on CRC cells. Based on these observations, we investigated whether a single exposure to 5-azanucleosides affects phosphoinositide 3-kinase (PI3-kinase)/Akt signaling, the main cellular pathway governing cell proliferation, survival, metabolism, and epithelial-mesenchymal transition [28, 29]. For this purpose, we examined the activation-associated phosphorylation of mammalian target of rapamycin (mTOR; Ser2481 - mainly associated with activation of mTOR complex 2 (mTORC2)), Akt (mTORC2-related phosphorylation of Ser473), and p70S6K (mTORC1-related phosphorylation of Thr389). DLD-1 cells were treated with 5-aza-dC (0.1–1 μM) and maintained in drug-free culture medium (the first passage after 3 days) for the next 6 or 20 days. As shown by Western blotting analysis, 5-aza-dC did not alter the expression of mTOR, Akt, and p70S6K proteins (Fig. 5c). Similarly, the level of phospho-mTOR remained unchanged 20 days after 5-aza-dC treatment. On the other hand, there was a dose-dependent reduction in Akt phosphorylation 20 days, but not 6 days, after 5-aza-dC treatment, which was accompanied by a reduction of downstream phospho-p70S6K.

**Fig. 6:**
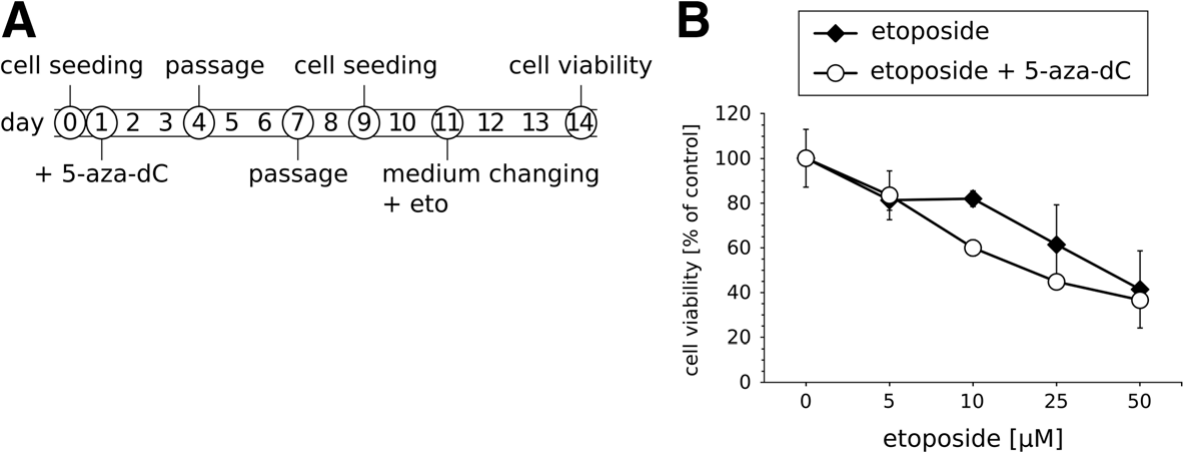
Pretreatment with 5-aza-dC sensitizes CRC cells to etoposide for a longer period of time. **a** Treatment scheme. **b** Cell viability of DLD-1 cells after sequential treatments with 1 μM 5-aza-dC and 5–50 μM etoposide (*n* = 4). Data are presented as means ± SD normalized to untreated control. Eto - etoposide

Since single treatments with 5-azanucleosides cause long-lasting effects on CRC cells, we investigated whether 5-azanucleosides could sensitize CRC cells to topoisomerase inhibitors for a longer period of time. DLD-1 cells were treated with 5-aza-dC (1 μM) and maintained in drug-free culture medium (the first passage after 3 days) followed by etoposide (5–50 μM) treatment 10 days after 5-aza-dC addition (Fig. 6a). This sequential treatment was still effective in reducing cell viability compared to each compound alone (Fig. 6b).

### Combinatorial treatments with 5-azanucleosides and topoisomerase inhibitors reduce colony-forming ability of HCT116 and DLD-1 cells

**Fig. 7:**
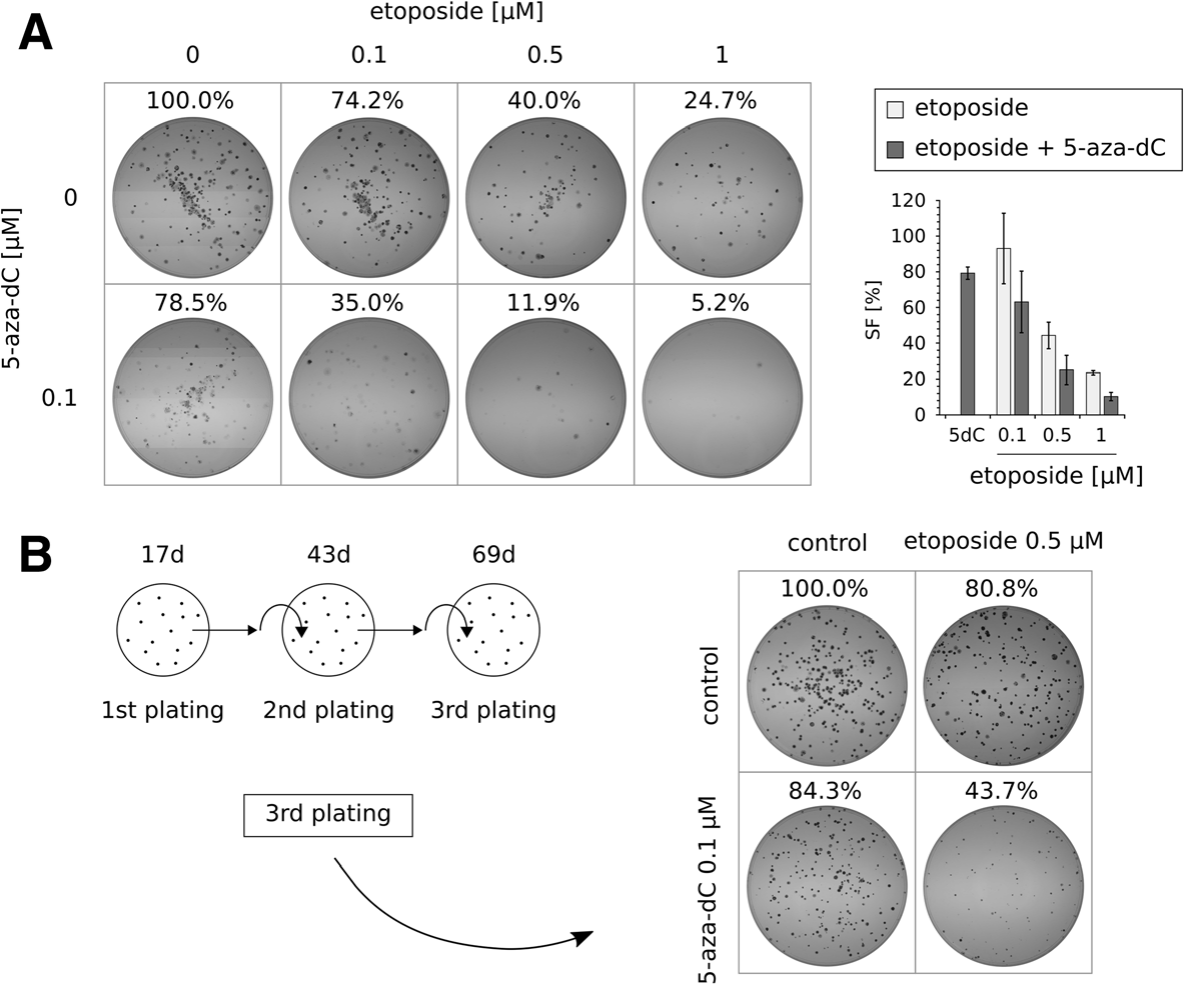
Combinatorial treatments reduce colony-forming ability of CRC cells. **a** Long-term colony formation assay. On the left: representative images of colonies formed by DLD-1 cells after sequential treatments with 0.1 μM 5-aza-dC and 0.1–1 μM etoposide. On the right: the surviving fraction (SF) of DLD-1 cells after sequential treatments with 0.1 μM 5-aza-dC and 0.1–1 μM etoposide (*n* = 3). **b** Serial replating assay. On the left: schematic representation of the assay. The experiment ended on day 69 since the cells were treated with 0.1 μM 5-aza-dC. On the right: representative images of colonies formed by DLD-1 cells in the third plating after sequential treatments with 0.1 μM 5-aza-dC and 0.5 μM etoposide. D - days

It has already been described that 5-azanucleosides decrease clonogenic potential of both solid tumor and leukemia cell lines [30, 31], therefore we investigated whether combinatorial DNA demethylating agents and topoisomerase inhibitors treatments could impair CRC cell colony-forming ability more effectively than those of individual compounds. For this purpose, we performed long-term colony formation assay. In DLD-1 cells, low-dose etoposide (0.1–1 μM) alone reduced the surviving fraction (SF) of cells in a concentration-dependent manner, whereas low-dose 5-aza-dC (0.1 μM) alone caused a 21.5% reduction in cell clonogenicity (Fig. 7a). Higher concentrations of each compound (5 μM etoposide and 1 μM 5-aza-dC) resulted in complete inhibition of colony formation (Additional file 6: Figure S5). However, the sequential treatments with low doses of 5-aza-dC and etoposide allowed to achieve similar strong inhibitory effect (Fig. 7a). The same inhibitory effects were observed in HCT116 cells (data not shown).

Furthermore, we examined the clonogenic growth of DLD-1 cells in serial replating assay, where the cells were treated with compounds only once, 69 days (5-aza-dC) and 68 days (etoposide) before the end of the experiment. In the third plating, low-dose etoposide (0.5 μM) and low-dose 5-aza-dC (0.1 μM) alone reduced the SF of cells by 19.2 and 15.7%, respectively (Fig. 7b). However, the combinatorial treatment caused a 56.3% reduction in cell clonogenic/replating ability, and the colonies were much smaller than either those formed by control or single-agent-treated cells.

## Discussion

The prognosis for patients with CRC, especially those with metastatic CRC, remains poor. Considering the limited effectiveness of the current chemotherapy (mainly due to both intrinsic and acquired drug resistance and dose-limiting side effects), new efficient drug combinations are urgently needed. In this study, we report that pretreatment of CRC cells with 5-azanucleosides at clinically relevant concentrations (0.1–1 μM 5-aza-dC and 4 μM 5-aza-C) enhances the anticancer effects of topoisomerase inhibitors, such as irinotecan, a topoisomerase I inhibitor commonly used in CRC treatment, as well as etoposide, doxorubicin, and mitoxantrone, topoisomerase II inhibitors approved for the treatment of other cancers. We found that prior exposure to 5-azanucleosides sensitized CRC cells to topoisomerase inhibitors, reducing cell viability and clonogenicity and increasing programmed cell death more effectively than individual compounds at the same or even higher concentrations. In our experimental settings, 5-azanucleosides did not cause considerable immediate toxic effects on CRC cells. HCT116 cells appeared to be the most sensitive of all tested cell lines to 5-azanucleosides, which is consistent with the previous report [32]. Topoisomerase inhibitors operate by stabilizing the covalent DNA-topoisomerase cleavage complexes, thereby generating DNA single- and double-strand breaks and ultimately leading to apoptosis [33, 34]. The mechanism of the mutual action of 5-azanucleosides and topoisomerase inhibitors still remains unclear, but it involves neither DNA damage accumulation nor ER stress augmentation, since we did not observe any increases in H2A.X phosphorylation and CHOP expression after combinatorial treatments. On the contrary, there was even a decrease in γH2A.X levels in HCT116 cells, which could possibly be explained by interference with H2A.X-associated DNA damage response pathway (for instance γH2A.X formation and stability), as 5-azanucleosides are obviously DNA affecting agents. This would lead to an insufficient DNA damage response and repair after combinatorial treatments and consequently increased cytotoxicity. Furthermore, a decrease in γH2A.X levels in HCT116 cells could be explained by reexpression of some epigenetically silenced genes encoding DNA damage response and repair proteins involved in homologous recombination (HR), non-homologous end joining (NHEJ), and/or mismatch repair (MMR) pathways [35, 36]. Interestingly, reexpression of these proteins can also sensitize CRC cells to DNA damaging agents. It was reported that 5-aza-dC-induced reexpression of Mlh1, a MMR protein, restores sensitivity of SW48 CRC cells to 5-fluorouracil [37]. This reexpression could be even more extensive after combinatorial treatments, since topoisomerase II was shown to regulate the maintenance of DNA methylation [38].

Not all CRC cell lines used in this study responded to the combinatorial treatments to the same extent, which may partially result from differential metabolism of 5-azanucleosides [39]. Yet the response to chemotherapy is mainly determined by molecular features of cancer cells [40]. Using the DLD-1/DKs-8 isogenic cell line pair, we established that the presence of the KRas mutation (p.G13D) is irrelevant in sequential 5-azanucleosides and topoisomerase inhibitors treatments. It can be hypothesized that the B-Raf mutation (p.V600E) may possibly contribute to resistance of CRC cells to the combinatorial treatments, since B-Raf-mutated HT-29 cells appeared to be the least sensitive of all tested cell lines to the combinatorial treatments. However, this issue definitely requires more research. Although it was reported that p53 mutations can be a prerequisite for 5-aza-C-induced sensitization to SN-38, an active metabolite of irinotecan [41], we found that 5-azanucleosides sensitize both p53-mutated and wild-type (wt) p53-expressing CRC cells to topoisomerase inhibitors (Table 1). On the other hand, Plumb et al. showed that 5-aza-dC-induced sensitization of human colon cancer xenografts to epirubicin, a topoisomerase II inhibitor, depends on reexpression of *MLH1* gene [42], while our results indicate that 5-azanucleosides induce sensitization to topoisomerase inhibitors in MMR-deficient CRC cells (HCT116, DLD-1; Table 1). Demethylation and reexpression of some apoptosis-related genes was also pointed out to be responsible for 5-aza-dC-induced sensitization to irinotecan [43]. In addition, genomic instability phenotypes (such as chromosomal instability (CIN) and microsatellite instability (MSI)) may possibly play a role in susceptibility to sequential 5-azanucleosides and topoisomerase inhibitors treatments, since HCT116 and DLD-1 cells are characterized as CIN-negative and MSI, whereas HT-29 cells as CIN-positive and microsatellite stable (MSS) [44].

The most important outcome of our study is that a single treatment with 5-azanucleosides is sufficient to induce long-lasting sensitization to topoisomerase inhibitors in CRC cells, effectively impairing their colony-forming ability in a sequential setting. We found that the combinatorial, but not separate, treatment with low doses of 5-aza-dC (0.1 μM) and etoposide (0.5 μM) caused a long-lasting (almost 70 days) reduction in clonogenic/replating ability of DLD-1 cells. Therefore, the sequential treatments with 5-azanucleosides and topoisomerase inhibitors have a therapeutic potential for CRC treatment, because targeting a clonogenic/tumor-initiating/stem cell-like subset of cancer cells is thought to be essential for a successful cancer therapy [45]. Since simultaneous treatments with 5-azanucleosides and topoisomerase inhibitors were ineffective in CRC cells (data not shown), epigenetic events are probably crucial in 5-azanucleosides-induced sensitization to topoisomerase inhibitors. In this study, CRC cells were pretreated with 5-azanucleosides before exposure to topoisomerase inhibitors in order to allow the cells to divide at least twice, which is required for passive loss of cytosine methylation to become permanent [6]. The sensitization to topoisomerase inhibitors may result from: i) reexpression of epigenetically silenced tumor suppressor genes thereby restoring proliferation control and apoptosis sensitivity; ii) relaxation of chromatin structure thereby facilitating access of inhibitors to topoisomerases; iii) enhancement of genomic instability; and/or iv) loss of DNMTs functions itself. Regardless of the direct mechanism involved, we noted that 5-azanucleosides exert long-lasting effects on CRC cells, reducing cell viability, proliferation, and changing cell morphology. In accordance with these observations, we found that a single exposure to 5-azanucleosides affects PI3-kinase/Akt signaling pathway. There was a considerable dose-dependent reduction in Akt phosphorylation 20 days after a single 5-aza-dC treatment. Akt phosphorylation on Ser473 can be mediated by DNA-dependent protein kinase (DNA-PK) or mTORC2, which regulates cell metabolism and cytoskeleton organization [46, 47]. The level of mTOR autophosphorylation (associated with activation of mTORC2) remained unchanged at that time point, which exclude its involvement in attenuation of the PI3-kinase/Akt signaling. On the other hand, it may result from 5-azanucleosides-induced enhanced activity of PTEN, the major negative regulator of PI3-kinase/Akt pathway. Interestingly, PTEN was reported to contribute to sensitization of cancer cells to various chemotherapeutic drugs [48, 49]. Decrease in Akt phosphorylation was accompanied by a profound reduction of downstream phospho-p70S6K, suggesting an impairment of mTORC1 activity. Disturbances in both mTORC1 and mTORC2 signaling pathways may possibly contribute to the observed changes in CRC cell morphology and decreases in cell viability and proliferation after 5-azanucleosides treatment.

## Conclusions

Taken together, we show that pretreatment of CRC cells with 5-azanucleosides potentiates the anticancer effects of topoisomerase inhibitors, suggesting that the combination of these two drug classes represents a promising therapeutic approach for the treatment of CRC and possibly other cancers. Importantly, prior exposure to 5-azanucleosides could potentially reduce topoisomerase inhibitors dosing and therefore decrease their side effects, such as myelosuppression. Similarly, it has been reported that 5-aza-C potentiates anticancer activity of cisplatin and, at the same time, attenuates the cisplatin-induced nephrotoxicity [50]. Thus, our findings strongly encourage future *in vivo* studies on combinatorial use of DNA demethylating agents and topoisomerase inhibitors.

## Additional files

Additional file 1: Table S1. Comprehensive summary of CRC cell viability results after sequential treatments with 5-azanucleosides and topoisomerase inhibitors. Data are presented as means ± SD normalized to untreated control. **P* < 0.05 compared with DNA demethylating agent treatment group and topoisomerase inhibitor treatment group. Combinatorial Index (CI) values for each drug combination were determined. N/D - no data. (PDF 45.3 kb)
Additional file 2: Figure S1. Pretreatment with 5-aza-dC enhances the cytotoxicity of etoposide in DKs-8 cells. Cell viability of DKs-8 cells after sequential treatments with 1 μM 5-aza-dC and 5-50 μM etoposide. Figure 1a shows the treatment scheme. Data are presented as means ± SD normalized to untreated control. **P* < 0.05 compared with DNA demethylating agent treatment group and topoisomerase inhibitor treatment group. (PDF 33.6 kb)
Additional file 3: Figure S2. Combinatorial treatments increase apoptosis-associated DNA fragmentation in CRC cells. Representative histograms of HCT116, DLD-1, and HT-29 cells after sequential treatments with 1 μM 5-aza-dC and 5-50 μM etoposide. Figure 1a shows the treatment scheme. (PDF 208 kb)
Additional file 4: Figure S3. Combinatorial treatments increase CRC cell apoptosis. Annexin V-FITC/PI-double staining of HCT116, DLD-1, and HT-29 cells after sequential treatments with 1 μM 5-aza-dC and 25 μM etoposide (*n* = 3). Figure 1a shows the treatment scheme. Data are presented as means ± SD. **P* < 0.05 compared with DNA demethylating agent treatment group and topoisomerase inhibitor treatment group. (PDF 177 kb)
Additional file 5: Figure S4. 5-Aza-dC reduces CRC cell proliferation. Cell proliferation of DLD-1 cells within 7 days of exposure to 1 μM 5-aza-dC alone. Green histograms: tested cells; white histograms: unstained cells; grey histograms: the cells stained on the day of analysis. The percentage of cells in gates represents non-proliferating cells. Cell proliferation was assessed by CellTrace Far Red Cell Proliferation Kit (Molecular Probes, Thermo Fisher Scientific) according to the manufacturer’s protocol. Briefly, the cells were stained with CellTrace Far Red (1 μM) for 20 min at 37 °C. Data was acquired on a FACSCalibur flow cytometer (Becton Dickinson, Franklin Lakes, NJ, USA) and analyzed using Flowing Software 2.5.1 software (Perttu Terho, Turku, Finland). (PDF 46 kb)
Additional file 6: Figure S5. Etoposide or 5-aza-dC treatment reduces colony-forming ability of CRC cells. Representative images of DLD-1 colonies after treatment with 5 μM etoposide or 1 μM 5-aza-dC. (PDF 927 kb)

## Abbreviations

5-aza-C: 5-azacytidine
5-aza-dC: 5-aza-2′-deoxycytidine
ACF: Aberrant crypt foci
AML: Acute myeloid leukemia
BSA: Bovine serum albumin
CHOP: CCAAT/enhancer-binding protein (C/EBP) homologous protein
CI: Combination index
CIN: Chromosomal instability
CRC: Colorectal cancer
DMEM: Dulbecco’s modified Eagle’s medium
DMSO: Dimethyl sulfoxide
DNA: Deoxyribonucleic acid
DNA-PK: DNA-dependent protein kinase
DNMT: DNA (cytosine-5) methyltransferase
Dox: Doxorubicin
ER: Endoplasmic reticulum
Eto: Etoposide
FBS: Fetal bovine serum
FDA: Food and Drug Administration
FITC: Fluorescein isothiocyanate
HR: Homologous recombination
HRP: Horseradish peroxidase
Irin: Irinotecan
MDS: Myelodysplastic syndromes
Mit: Mitoxantrone
MLH1: MutL homolog 1
MMR: Mismatch repair
MSH3: MutS homolog 3
MSH6: MutS homolog 6
MSI: Microsatellite instability
MSS: Microsatellite stable
mTOR: Mammalian target of rapamycin
NHEJ: Non-homologous end joining
NP-40/Nonidet P-40: 4-nonylphenyl-polyethylene glycol
PBS: Phosphate-buffered saline
PE: Plating efficiency
PI: Propidium iodide
PI3-kinase: Phosphoinositide 3-kinase
PVDF: Polyvinylidene difluoride
RIPA: Radioimmunoprecipitation assay
RNase A: Ribonuclease A
RT: Room temperature
SD: Standard deviation
SDS: Sodium dodecyl sulfate
SDS-PAGE: SDS-polyacrylamide gel electrophoresis
SF: Surviving fraction
TBS: Tris-buffered saline
Tris: Tris(hydroxymethyl)aminomethane
Wt: Wild-type
