# Supplementary material for: Long-lasting reduction in clonogenic potential of colorectal cancer cells by sequential treatments with 5-azanucleosides and topoisomerase inhibitors
Source: BMC Cancer. 2016 Nov 16;16:893. doi: 10.1186/s12885-016-2925-6 (PMC5112712; doi:10.1186/s12885-016-2925-6)
Supplement: Additional file 1: Table S1. — Comprehensive summary of CRC cell viability results after sequential treatments with 5-azanucleosides and topoisomerase inhibitors. Data are presented as means ± SD normalized to untreated control. *P < 0.05 compared with DNA demethylating agent treatment group and topoisomerase inhibitor treatment group. Combinatorial Index (CI) values for each drug combination were determined. N/D - no data. (PDF 45.3 kb) [file 12885_2016_2925_MOESM1_ESM.pdf]

5-aza-dC

HCT116

	conc. (µM)	% of control ± SD	+ 5-aza-dC (1 µM)	CI
irinotecan	0	100,0 ± 4,3	67,6 ± 12,8	
	5	83,8 ± 10,3	55,2 ± 11,7	0,22
	10	81,4 ± 8,0	47,4 ± 8,5	0,16
	25	68,9 ± 6,4	36,7 ± 4,9 *	0,13
	50	58,4 ± 6,0	28,3 ± 3,1 *	0,12
	n=4			
etoposide	0	100,0 ± 7,6	64,2 ± 5,0	
	5	70,5 ± 3,9	50,1 ± 3,2 *	0,16
	10	66,2 ± 2,7	46,3 ± 1,7 *	0,16
	25	63,8 ± 5,4	38,8 ± 3,4 *	0,13
	50	49,6 ± 1,8	34,2 ± 3,1 *	0,13
	n=4			
doxorubicin	0,00	100,0 ± 2,5	58,3 ± 3,6	
	0,05	84,4 ± 9,5	48,2 ± 4,0 *	0,19
	0,10	70,3 ± 2,0	41,7 ± 1,0 *	0,20
	0,25	62,1 ± 3,3	31,2 ± 3,1 *	0,22
	0,50	46,5 ± 7,2	20,7 ± 1,0 *	0,20
	n=3			
mitoxantrone	0,00	100,0 ± 2,4	56,9 ± 5,3	
	0,05	69,1 ± 10,0	52,5 ± 13,2	0,12
	0,10	66,5 ± 10,4	50,2 ± 13,1	0,10
	0,25	63,6 ± 11,9	39,0 ± 10,3 *	0,04
	0,50	61,7 ± 4,0	25,1 ± 3,4 *	0,01
	n=4			

DLD-1

	conc. (µM)	% of control ± SD	+ 5-aza-dC (1 µM)	CI
irinotecan	0	100,0 ± 7,0	80,8 ± 5,0	
	10	104,1 ± 2,8	79,6 ± 4,2	0,35
	25	102,4 ± 7,8	77,7 ± 3,7	0,52
	50	85,0 ± 6,8	54,3 ± 7,4 *	0,55
	75	64,8 ± 9,1	34,0 ± 3,3 *	0,62
	n=3			
etoposide	0	100,0 ± 6,5	86,3 ± 9,5	
	5	89,3 ± 5,5	76,7 ± 5,4	0,43
	10	88,1 ± 6,4	63,8 ± 6,2 *	0,33
	25	77,4 ± 4,4	32,5 ± 1,0 *	0,17
	50	49,2 ± 7,0	27,5 ± 3,0 *	0,27
	n=5			
doxorubicin	0,0	100,0 ± 4,9	86,0 ± 5,2	
	0,1	86,0 ± 7,5	70,8 ± 8,9 *	0,38
	0,3	84,5 ± 7,3	53,6 ± 8,6 *	0,44
	0,6	60,6 ± 4,4	31,9 ± 7,7 *	0,35
	0,9	38,2 ± 5,7	21,5 ± 2,3 *	0,30
	n=4			
mitoxantrone	0,00	100,0 ± 4,8	85,9 ± 3,2	
	0,05	83,7 ± 4,9	72,3 ± 4,4 *	0,20
	0,10	84,2 ± 11,8	67,1 ± 9,5	0,16
	0,25	78,2 ± 12,0	52,1 ± 12,2	0,06
	0,50	70,9 ± 5,0	33,1 ± 7,1 *	0,01
	n=3			

DKs-8

	conc. (µM)	% of control ± SD	+ 5-aza-dC (1 µM)
irinotecan	0	100,0 ± 4,4	92,1 ± 6,1
	10	96,3 ± 7,2	78,9 ± 3,8 *
	25	91,0 ± 6,6	70,4 ± 7,1 *
	50	65,9 ± 14,2	37,9 ± 3,6
	75	32,8 ± 2,5	26,8 ± 4,1
	n=3		
etoposide	0	100,0 ± 3,0	91,6 ± 4,9
	5	94,3 ± 6,8	81,3 ± 5,6 *
	10	95,5 ± 7,4	74,7 ± 6,7 *
	25	82,3 ± 7,0	57,2 ± 10,1 *
	50	60,0 ± 11,0	35,9 ± 6,4 *
	n=4		
doxorubicin	N/D		
mitoxantrone	N/D		

HT-29

	conc. (µM)	% of control ± SD	+ 5-aza-dC (1 µM)	CI
irinotecan	0	100,0 ± 2,8	78,7 ± 5,4	
	5	90,0 ± 6,9	70,6 ± 9,8	0,36
	10	83,6 ± 6,3	65,3 ± 7,9	0,57
	25	54,1 ± 2,6	47,9 ± 6,2	0,80
	50	33,2 ± 3,6	26,3 ± 0,5	0,77
	n=3			
etoposide	0	100,0 ± 5,9	88,1 ± 11,9	
	5	96,6 ± 8,5	82,4 ± 10,1	0,40
	10	87,8 ± 5,6	77,4 ± 12,9	0,61
	25	67,3 ± 5,1	57,0 ± 11,9	0,81
	50	38,4 ± 6,3	34,5 ± 1,8	0,91
	n=3			
doxorubicin	0,0	100,0 ± 5,8	99,7 ± 5,4	
	0,1	91,0 ± 2,7	94,2 ± 9,1	1,95
	0,3	89,6 ± 4,2	88,6 ± 6,7	1,51
	0,6	82,1 ± 7,9	79,8 ± 6,0	0,89
	0,9	74,0 ± 10,8	61,4 ± 4,9	0,27
	n=3			
mitoxantrone	0,00	100,0 ± 2,9	100,7 ± 4,9	
	0,10	88,1 ± 3,9	88,2 ± 8,5	1,11
	0,25	76,4 ± 5,7	76,9 ± 8,7	0,98
	0,50	69,1 ± 14,1	69,6 ± 5,6	1,22
	1,00	51,8 ± 5,5	48,8 ± 8,9	0,82
	n=3			

5-aza-C

HCT116

	conc. (µM)	% of control ± SD	+ 5-aza-C (4 µM)	CI
irinotecan	0	100,0 ± 4,3	77,7 ± 6,2	
	5	93,7 ± 5,4	71,2 ± 7,7	0,99
	10	85,2 ± 6,8	62,5 ± 5,9 *	0,70
	25	75,1 ± 6,2	49,7 ± 4,6 *	0,52
	50	65,0 ± 3,9	33,3 ± 0,8 *	0,35
	n=3			
etoposide	0	100,0 ± 4,4	76,9 ± 12,7	
	5	87,0 ± 14,5	68,4 ± 8,5	0,75
	10	83,9 ± 11,6	65,0 ± 10,8	0,65
	25	81,6 ± 15,7	60,4 ± 11,1	0,55
	50	71,5 ± 17,4	50,6 ± 11,5	0,34
	n=4			
doxorubicin	0	100,0 ± 4,9	74,5 ± 10,8	
	0,05	98,7 ± 10,9	69,8 ± 11,7	0,93
	0,10	86,4 ± 14,9	59,6 ± 7,7	0,68
	0,25	78,6 ± 9,8	46,3 ± 10,6 *	0,67
	0,50	53,8 ± 8,1	26,3 ± 5,8 *	0,60
	n=4			
mitoxantrone	0,00	100,0 ± 4,4	70,3 ± 11,6	
	0,05	80,8 ± 4,1	64,1 ± 11,3	0,65
	0,10	79,5 ± 5,8	60,9 ± 8,3	0,60
	0,25	74,9 ± 8,7	50,6 ± 7,3	0,40
	0,50	60,1 ± 2,8	31,1 ± 6,3 *	0,13
	n=4			

DLD-1

	conc. (µM)	% of control ± SD	+ 5-aza-C (4 µM)	CI
irinotecan	0	100,0 ± 2,8	91,6 ± 5,4	
	10	95,0 ± 5,6	78,0 ± 3,8 *	0,40
	25	89,5 ± 6,7	63,0 ± 4,9 *	0,42
	50	74,0 ± 8,9	41,4 ± 7,9 *	0,39
	75	56,8 ± 4,8	26,3 ± 4,3 *	0,34
	n=3			
etoposide	0	100,0 ± 4,0	85,1 ± 2,2	
	5	88,6 ± 3,5	70,6 ± 4,9 *	0,32
	10	80,4 ± 5,8	62,4 ± 6,0 *	0,34
	25	77,3 ± 10,2	39,6 ± 1,3 *	0,24
	50	49,7 ± 15,4	28,2 ± 6,6	0,24
	n=3			
doxorubicin	0,00	100,0 ± 4,9	83,8 ± 8,4	
	0,10	86,0 ± 7,5	74,6 ± 6,2	0,47
	0,30	84,5 ± 7,3	53,5 ± 8,4 *	0,46
	0,60	60,6 ± 4,4	29,2 ± 2,8 *	0,32
	0,90	38,2 ± 5,7	20,9 ± 1,2 *	0,30
	n=4			
mitoxantrone	0,00	100,0 ± 4,0	85,6 ± 2,9	
	0,05	86,1 ± 6,6	66,4 ± 3,3 *	0,31
	0,10	75,8 ± 10,0	60,3 ± 6,0	0,40
	0,25	73,3 ± 17,9	36,6 ± 2,5	0,27
	0,50	43,3 ± 3,8	25,3 ± 3,1 *	0,27
	n=3			

DKs-8

	conc. (µM)	% of control ± SD	+ 5-aza-C (4 µM)
irinotecan	0	100,0 ± 4,4	87,0 ± 3,1
	10	96,3 ± 7,2	70,3 ± 4,2 *
	25	91,0 ± 6,6	47,3 ± 1,1 *
	50	65,9 ± 14,2	28,0 ± 2,3
	75	32,8 ± 2,5	23,5 ± 3,3 *
	n=3		
etoposide	0	100,0 ± 3,5	89,5 ± 7,7
	5	96,9 ± 5,5	82,6 ± 5,2
	10	95,5 ± 7,4	76,2 ± 9,1
	25	87,8 ± 4,1	56,0 ± 10,3 *
	50	60,7 ± 4,6	31,4 ± 4,3 *
	n=3		
doxorubicin	N/D		
mitoxantrone	N/D		

HT-29

	conc. (µM)	% of control ± SD	+ 5-aza-C (4 µM)	CI
irinotecan	0	100,0 ± 2,8	70,1 ± 6,8	
	5	90,0 ± 6,9	66,9 ± 10,5	0,76
	10	83,6 ± 6,3	46,7 ± 12,7	0,35
	25	54,1 ± 2,6	26,5 ± 5,9 *	0,39
	50	33,2 ± 3,6	21,0 ± 2,0 *	0,61
	n=3			
etoposide	0	100,0 ± 4,8	88,8 ± 2,2	
	5	97,0 ± 9,0	77,9 ± 4,5	2,27
	10	93,2 ± 7,8	75,3 ± 1,4	1,77
	25	79,4 ± 15,2	68,3 ± 13,0	1,28
	50	53,4 ± 16,2	41,7 ± 10,9	0,70
	n=3			
doxorubicin	0,0	100,0 ± 6,1	91,1 ± 4,9	
	0,1	97,7 ± 6,9	86,6 ± 8,3	10,58
	0,3	96,4 ± 4,3	83,1 ± 5,5	5,46
	0,6	83,5 ± 6,8	63,6 ± 5,6 *	0,71
	0,9	73,4 ± 10,9	45,1 ± 8,6 *	0,37
	n=3			
mitoxantrone	0,00	100,0 ± 3,5	90,1 ± 3,9	
	0,10	92,7 ± 6,4	83,1 ± 3,2	5,34
	0,25	82,1 ± 8,9	69,8 ± 4,4	1,01
	0,50	85,4 ± 4,6	53,7 ± 4,9 *	0,41
	1,00	56,4 ± 6,3	35,2 ± 4,5 *	0,27
	n=3			
