# Supplementary figures and images for: Long-lasting reduction in clonogenic potential of colorectal cancer cells by sequential treatments with 5-azanucleosides and topoisomerase inhibitors
Source: BMC Cancer. 2016 Nov 16;16:893. doi: 10.1186/s12885-016-2925-6 (PMC5112712; doi:10.1186/s12885-016-2925-6)

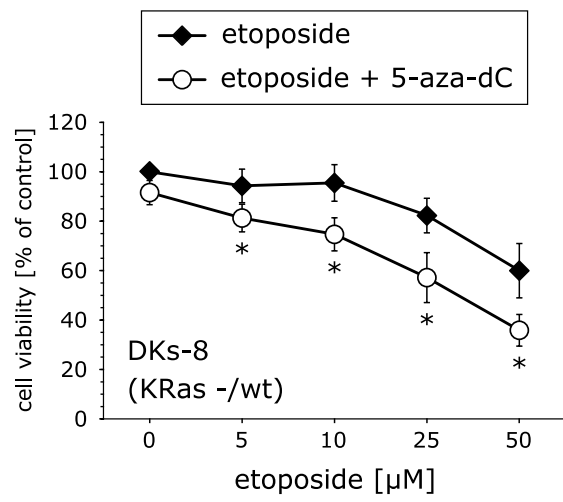

Supplement: Additional file 2: Figure S1. — Pretreatment with 5-aza-dC enhances the cytotoxicity of etoposide in DKs-8 cells. Cell viability of DKs-8 cells after sequential treatments with 1 μM 5-aza-dC and 5-50 μM etoposide. Figure 1a shows the treatment scheme. Data are presented as means ± SD normalized to untreated control. *P < 0.05 compared with DNA demethylating agent treatment group and topoisomerase inhibitor treatment group. (PDF 33.6 kb) [file 12885_2016_2925_MOESM2_ESM.pdf]

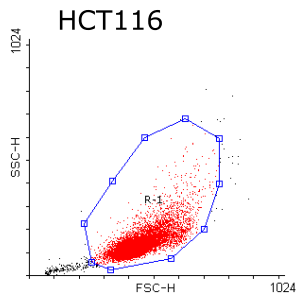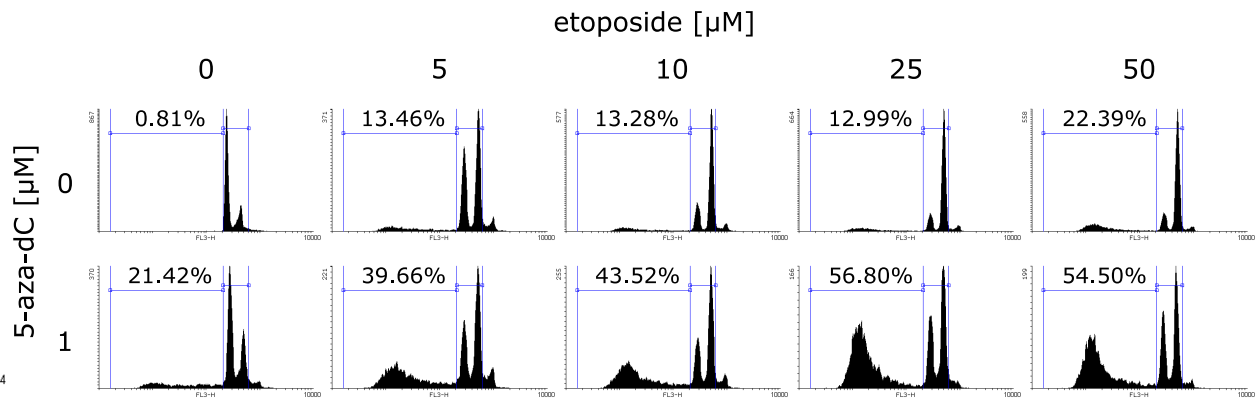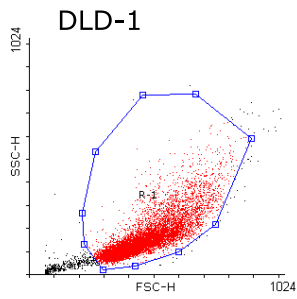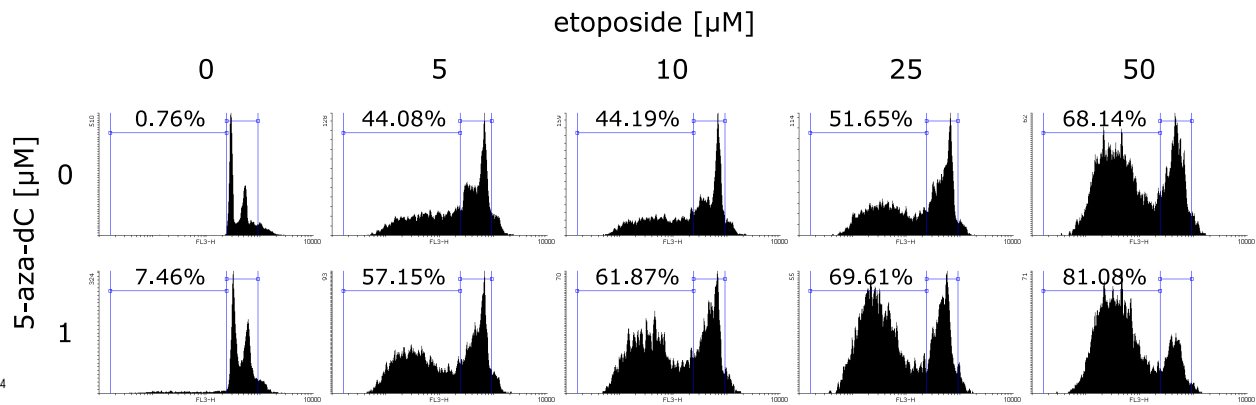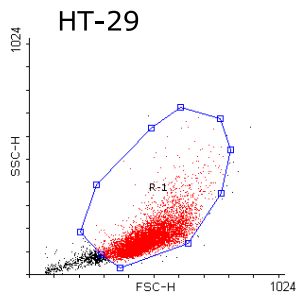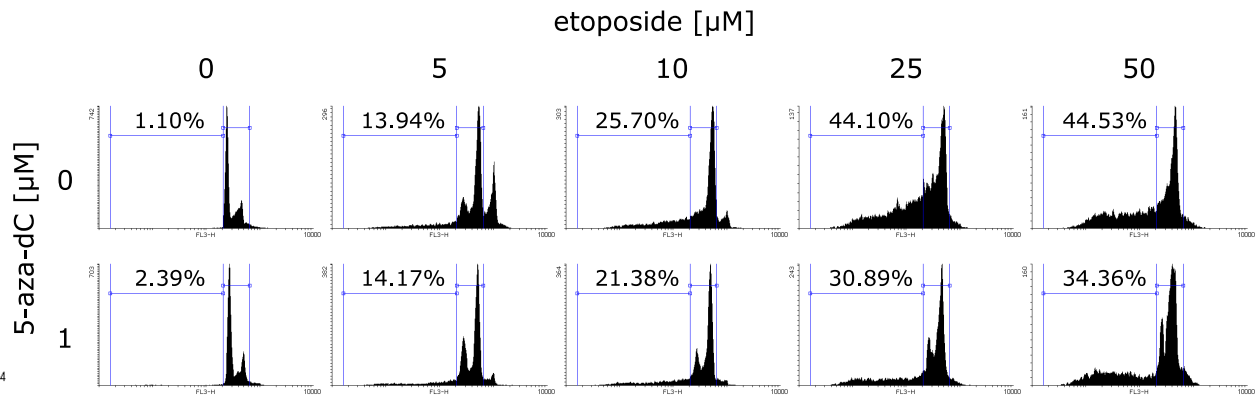

Supplement: Additional file 3: Figure S2. — Combinatorial treatments increase apoptosis-associated DNA fragmentation in CRC cells. Representative histograms of HCT116, DLD-1, and HT-29 cells after sequential treatments with 1 μM 5-aza-dC and 5-50 μM etoposide. Figure 1a shows the treatment scheme. (PDF 208 kb) [file 12885_2016_2925_MOESM3_ESM.pdf]

cell seeding    passage    cell proliferation

day (0) (1) 2 (3) (4) 5 6 7 (8)

+ 5-aza-dC    + CellTrace Far Red

control

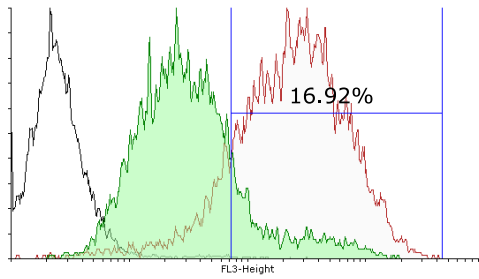

5-aza-dC 1  $\mu$ M

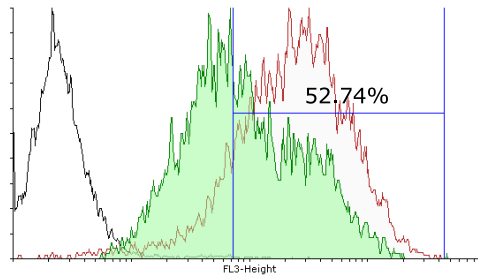

Supplement: Additional file 5: Figure S4. — 5-Aza-dC reduces CRC cell proliferation. Cell proliferation of DLD-1 cells within 7 days of exposure to 1 μM 5-aza-dC alone. Green histograms: tested cells; white histograms: unstained cells; grey histograms: the cells stained on the day of analysis. The percentage of cells in gates represents non-proliferating cells. Cell proliferation was assessed by CellTrace Far Red Cell Proliferation Kit (Molecular Probes, Thermo Fisher Scientific) according to the manufacturer’s protocol. Briefly, the cells were stained with CellTrace Far Red (1 μM) for 20 min at 37 °C. Data was acquired on a FACSCalibur flow cytometer (Becton Dickinson, Franklin Lakes, NJ, USA) and analyzed using Flowing Software 2.5.1 software (Perttu Terho, Turku, Finland). (PDF 46 kb) [file 12885_2016_2925_MOESM5_ESM.pdf]

etoposide [ $\mu\text{M}$ ]

0

5

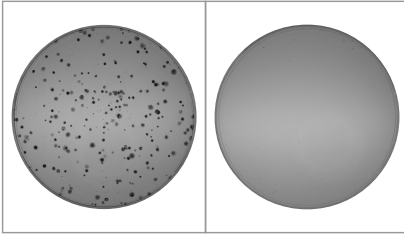

5-aza-dC [ $\mu\text{M}$ ]

0

1

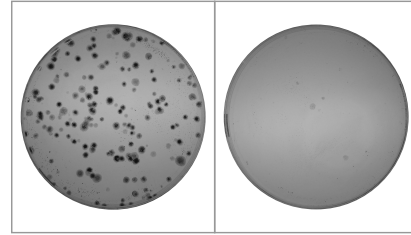

Supplement: Additional file 6: Figure S5. — Etoposide or 5-aza-dC treatment reduces colony-forming ability of CRC cells. Representative images of DLD-1 colonies after treatment with 5 μM etoposide or 1 μM 5-aza-dC. (PDF 927 kb) [file 12885_2016_2925_MOESM6_ESM.pdf]
