# Supplementary material for: Long-lasting reduction in clonogenic potential of colorectal cancer cells by sequential treatments with 5-azanucleosides and topoisomerase inhibitors
Source: BMC Cancer. 2016 Nov 16;16:893. doi: 10.1186/s12885-016-2925-6 (PMC5112712; doi:10.1186/s12885-016-2925-6)
Supplement: Additional file 4: Figure S3. — Combinatorial treatments increase CRC cell apoptosis. Annexin V-FITC/PI-double staining of HCT116, DLD-1, and HT-29 cells after sequential treatments with 1 μM 5-aza-dC and 25 μM etoposide (n = 3). Figure 1a shows the treatment scheme. Data are presented as means ± SD. *P < 0.05 compared with DNA demethylating agent treatment group and topoisomerase inhibitor treatment group. (PDF 177 kb) [file 12885_2016_2925_MOESM4_ESM.pdf]

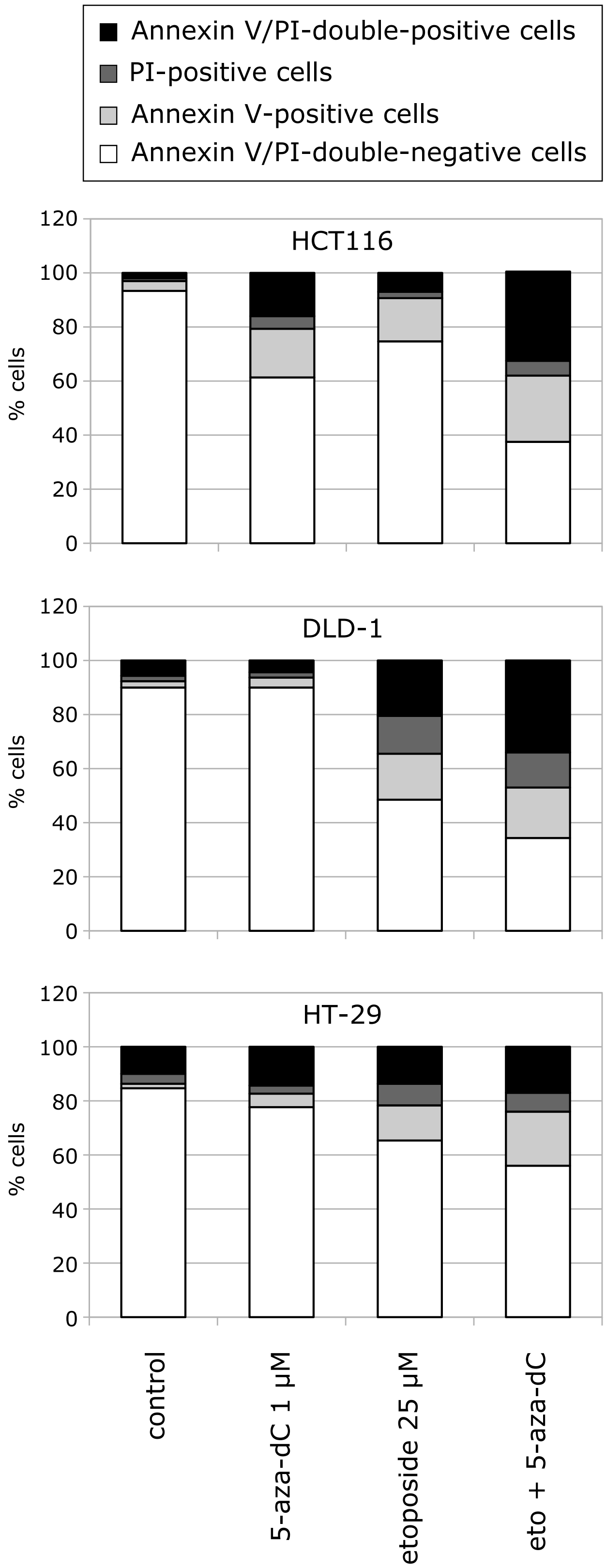


HCT116 — % cells ± SD

| | C | 5dC | eto | eto+5dC |
|---|---|---|---|---|
| Annexin V-/PI- | 93.3±0.6 | 61.3±10.1 | 74.7±4.5 | 37.5±0.7 |
| Annexin V+/PI- | 3.7±0.6 | 18.0±2.0 | 16.0±4.4 | 24.5±4.9 |
| Annexin V-/PI+ | 1.0±1.0 | 4.7±0.6 | 2.3±0.6 | 5.5±2.1 |
| Annexin V+/PI+ | 2.0±0.0 | 16.0±8.9 | 7.0±1.0 | 33.0±2.8 |

DLD-1 — % cells ± SD

| | C | 5dC | eto | eto+5dC |
|---|---|---|---|---|
| Annexin V-/PI- | 90.0±3.6 | 90.0±2.6 | 48.5±4.9 | 34.3±5.5 |
| Annexin V+/PI- | 2.3±2.3 | 3.7±1.2 | 17.0±1.4 | 18.7±4.2 |
| Annexin V-/PI+ | 2.0±1.0 | 2.0±1.0 | 14.0±7.1 | 13.0±5.3 |
| Annexin V+/PI+ | 5.7±1.2 | 4.3±0.6 | 20.5±0.7 | 34.0±3.6 |

HT-29 — % cells ± SD

| | C | 5dC | eto | eto+5dC |
|---|---|---|---|---|
| Annexin V-/PI- | 84.7±0.6 | 77.7±2.5 | 65.3±1.5 | 56.0±1.4 |
| Annexin V+/PI- | 1.7±2.1 | 5.0±3.0 | 13.0±4.6 | 20.0±4.2 |
| Annexin V-/PI+ | 3.7±2.1 | 3.0±1.7 | 8.0±4.4 | 7.0±1.4 |
| Annexin V+/PI+ | 10.0±2.0 | 14.3±1.5 | 13.7±3.2 | 17.0±4.2* |
